# Behind bars: the burden of being a woman in Brazilian prisons

**DOI:** 10.1186/s12914-020-00247-7

**Published:** 2020-10-29

**Authors:** Priscila França de Araújo, Ligia Regina Franco Sansigolo Kerr, Carl Kendall, George W. Rutherford, David W. Seal, Roberto da Justa Pires Neto, Patrícia Neyva da Costa Pinheiro, Marli Teresinha Gimeniz Galvão, Larissa Fortunato Araújo, Francisco Marto Leal Pinheiro, Ana Zaira da Silva

**Affiliations:** 1 Department of Public Health, Federal University of Ceara, Professor Costa Mendes, 1608 - Didactic Block, 5th floor Neighboor Rodolfo Teófilo, Fortaleza, Ceará 60.430-140 Brazil; 2 School of Public Health and Tropical Medicine Tulane University, New Orleans, USA; 3 University of California, San Francisco, USA

**Keywords:** Reproductive health, Prisons, Women, Community health

## Abstract

**Background:**

Brazil has the third largest prison population in the world. In 2016, the female prison population totaled 42,000, an increase of 656% over the population recorded in the early 2000s. The objective of this study was to describe the socialeconomic and reproductive health of women in Brazilian prisons, and the specific assistance received within the prison system.

**Methods:**

This is a first of its kind national survey conducted in 15 female prisons in eight Brazilian states between 2014 and 2015. The sample consisted of 1327 women in closed or semi-open prison regimes. Data collection used Audio Computer-Assisted Self-Interviewing (ACASI). STATA v.15. Was use in analysis. The study was submitted to the Research Ethics Committee of the Federal University of Ceará, under CEP protocol No. 1,024,053.

**Results:**

The population was overwhelmingly Black or Brown, poor and little educated. When women worked previously, they had worked as domestic servants and were the sole source of income for their families. Most were mothers, with 39% having children less than 10 years old, now in the care of others. Most were in jail for drug-related crimes. Prisons were crowded, with more than 2/3rds of the inmates sharing a cell with 6 or more inmates. Services were provide, but women had not had a cervical cancer screening within the past 3 years and breast cancer screening was not conducted.

**Conclusions:**

Overall, given their backround and prison conditions they are unlikely to change the circumstances that brought them to prison in the first place.

**Supplementary Information:**

The online version contains supplementary material available at 10.1186/s12914-020-00247-7.

## Background

It is estimated that the global prison population is 10.35 million. Brazil has the third largest prison population when considering those serving house-arrest sentences [1]. Although men comprise the majority in the prison system [2], it is estimated that there are more than 714,000 women in penal facilities worldwide [3]. The Brazilian female population incarcerated in 2014 was the fifth largest in the world. In 2016, the same population reached a total of 42,000, an increase of 656% over that recorded in the early 2000s. Such growth far exceeded that observed in the male population, of 293% [4].

This increase in female prisoners in Brazil brings to the foreground issues of gender equality, social costs, and on the health front, sexual and reproductive health. Poor prison infrastructure does not meet women’s needs, which include lack of toilets and basic hygiene supplies. Most female prisons were designed to house a male population, given their history and predominance in crimes and incarceration. The Brazilian prison system is known internationally as a human rights violator, and when this system tries to address the health of incarcerated women, it neglects a broad range of specific needs such as woman specialized services, family services, and psychological needs [5, 6]. Such conditions contribute to increasing vulnerability for women in prison and after leaving prison for a range of serious health problems [7].

Such problems were aggravated in the face of the current pandemic of Covid-19, where women with children under 12 years of age and pregnant women did not have their right granted to serve their sentence at home, remaining exposed to the virus through contact with prison staff who act as possible disease vectors. The visits of family members were also suspended, adding to the feeling of abandonment and deepening the issues of social vulnerability for prisoners and recently released women [8].

The National Policy of Attention to Women in Situations of Deprivation of Freedom and Egressed from the Prison System (2014) aims to improve state prison policies, improve the prison environment by integrating research and training for prison staff and professionals working in the prison system, and improve the quality of data on the female prison system in Brazil [9]. Women in the prison system are entitled to facilities and materials to meet their specific needs, for strategies aimed at the prevention, treatment, and care of diseases, including HIV/AIDS, and access to a physician for physical and mental assessment and treatment [10, 11].

There are few studies addressing women’s reproductive health in the Brazilian prison system. Thus, the present assessment, which used data from the first national health survey of female prisoners in Brazil, is a source of information for scholars, managers, and health professionals to plan and implement public policies directed towards the health of these women. Therefore, the objective of this study was to describe the socialeconomic and reproductive health of women in Brazilian prisons, and the specific assistance received within the prison system.

## Method

### Study design and location

This assessment is a cross-sectional study based on a National Health Survey of the female penitentiary population and prison employees conducted in eight Brazilian states and the Federal District (FD), which analyzed various aspects related to sexually transmitted infections, noncommunicable chronic diseases, violence, and the mental health of women in Brazilian prisons. The study was conducted from January 2014 to December 2015, in 15 female prison units located in the Brazilian North (Pará and Rondônia), Northeast (Ceará), Midwest (Federal District and Mato Grosso), Southeast (São Paulo and Minas Gerais), and South (Paraná and Rio Grande do Sul). In Brazil, there are about 146 prisons providing a closed regime, however, we chose the 15 establishments mentioned above due to their greater number of women prisoners, national representation, and because they have some type of health service within the unit [12].

One of the limitations of this study is the sample size. Some prisons created obstacles for participation in the survey. Selecting new prisons to replace them required a long negotiation process with the authorities responsible for the prisons in different federal, state, and municipal levels. Because the time to negotiate participation of these prisons was greatly extended, the study lasted longer than expected and the financial resources planned for the complete sample were no longer available. This resulted in a smaller final sample size than we orginally planned. Moreover, the state that was most affected in the sample was São Paulo, which has the largest prison population in the country. First, we tried to lessen the effect of these obstacles. For this, we selected three prisons, instead of the six prisons initially identified, and we chose a large, medium and small prison to be more representative of the state’s prison population. To identify ways that this decision could have biased our outcome, we compared our study data for HIV seroprevalance for the state with results from a study conducted a year earlier by the state of São Paulo in all female prisons. We found that the results were not significantly different.

### Population and sample

The sample consisted of women in the Brazilian prison system who had been in closed or semi-open prison regimes. Exclusion criteria included: women who did not speak Portuguese, who were uncommunicative due to mental health issues, or who were absent from the prison or cell block on the day of interview.

The selection of states by region was intentional, given that they contained the largest female prison populations [2]. Next, the prisons were stratified according to location (capital, metropolitan region, or interior). In order to be included in the sample, the prisons needed to provide health services and hold more than 75 inmates. The minimum sample size was estimated at 2518 residents (based on anticipated STI rates and standard criteria for precision). Increasing the sample population by 10% for unanticipated sampling or data collection errors, final sample size was calculted to be 2714 women. We encountered both administrative and financial obstacles, however, and the final sample consisted of 1327 women.

### Data collection

The data were collected using computer-assisted self-interview (Audio Computer-Assisted Self-Interviewing - ACASI), appropriate for exploring personal and sensitive issues in the the prison setting. Each participant was previously informed about the research objectives and then agreeing to participate received a tablet containing a letter of consent to sign, as well as the closed-ended questionnaire for the survey. This questionnaire was developed by the researchers specifically for this study, covering different themes on women’s prison health. We felt that the ACASI would ensure the confidentiality of responses and enhance reliability and validity of the research. However, perhaps due to the low educational level and difficulties in handling the tablet, the majority of respondents requested that they be interviewed by the researchers.

Variables of interest included socioeconomic variables such as age, level of education and number of children, income, whether or not the individual was a beneficiary of government programs, housing and living conditions, and prison history, including number of incarcerations and time spent in prison and types of crime. Race, following standard Brazilian practice (black, brown, white, yellow, and indigenous) was self-reported [13] The variables related to reproductive health included: age at debut and first pregnancy as recommended [14], menstrual status, access to health services, and preventive strategies and symptoms for sexually transmitted infections (STI).

### Data analysis

Analyses were performed using the complex analysis module in STATA®v.15. The sample was weighted according to the sampling design, with the weight being the inverse of the product of the probabilities of the sampling units at each of the stages of the sample design. Initial data coding and cleaning was conducted in SPSS® v 20.0. Variables of interest with 95% confidence intervals were estimated.

### Ethical aspects

The study followed the recommendations of Resolution 466/12 of the National Health Council (CNS) and was submitted to the Research Ethics Committee of the Federal University of Ceará, under CEP protocol No. 1,024,053 and approved on January 30, 2013.

Especially given the nature of research in prisons we and our IRB were concerned that the four basic principles of bioethics: autonomy, non-maleficence, beneficence and justice, were respected. That required in data collection a private environment to respond to the questionnaire, and anonymity for our participants, which we provided. All study participants signed the Free and Informed Consent Form.

## Results

The total sample of women was 1327. Most prisoners (75.6%) were under 40 years old, self-reported being Black or Brown (65.1%; 95%CI: 60.3–70.1). Less than on fourth reported having a regular male partner (22.5%; 95%CI: 19.7–24.5), one fourth having a regular female partner (24.5%; 95%CI: 22.1–27.2); and married or in stable union (10.0%; 95%CI: 8.4–11.9). Most reported as catholic (41.6%; 95%CI: 38.8–44.5), with incomplete primary education (45.2%; 95%CI: 42.4–48.1), and did not study in prison (71.2%; 95%CI: 68.8–73.5). Approximately 2/3 of the female prisoners (68.1%; 95%CI: 65.5–70.6) were repeat offenders, with drug trafficking being the most frequent cause of incarceration (65.6%; 95%CI: 62.9–68.3). Just under half of them (41.4%; 95%CI: 38.6–14.1) received remuneration for labor in prison, while around 3/4 (74.5%; 95%CI: 70.4–78.8) worked prior to being incarcerated, with domestic service (31.8%; 95%CI: 27.4–36.5) being the main activity, and 36.5% (95%CI: 33.7–39.3) were the primary sources of income for their families. As for prison conditions, about a third occupied cells with less than 5 inmates (31.9, 95%IC: 30.2–33.5) and 35% (95%CI: 32.9–37.2) shared their cell with 11–19 people and received few visits, with mothers (33.2%; 95%CI: 30.5–36.0) being the most frequent. The overwhelming majority did not receive intimate visits in the prison (90%; 95%CI: 88.2–91.5) (Table 1).

**Table 1:** Sociodemographic and prison characteristics of women incarcerated in Brazil, 2018

Characteristics	%	95% CI
**Age group in years (*N*** **= 1327)**		
18–19	0.4	0.2–0.9
21–24	16.5	14.5–18.8
25–29	25.4	22.9–28.0
30–39	33.3	30.6–36.1
40–49	15.4	13.4–17.7
> 50	8.7	7.1–10.5
**Race (*N*** **= 1318)**		
Black	15.3	13.3–17.6
Brown	49.8	47.0–52.5
White	31.5	28.9–34.2
Yellow	2.4	1.7–3.4
Indigenous	1.0	0.6–1.7
**Marital status (*N*** **= 1325)**		
Single without a regular partner	43.5	40.7–46.3
Has regular male partner	22.0	19.7–24.5
Has regular female partner	24.5	22.1–27.2
Married or stable union	10.0	8.4–11.9
**Religion (*N*** **= 1313)**		
Has no religion or belief	14.5	12.6–16.6
Catholic	41.6	38.8–44.5
Evangelical	37.8	35–40.6
Spiritualist	5.6	4.3–7.2
Other	0.6	0.2–1.3
**Education (*N*** **= 1324)**		
Illiterate	3.0	2.1–4.1
Incomplete primary education	45.2	42.4–48.1
Complete primary education and incomplete middle school	33.1	30.4–35.9
Complete middle school and incomplete university education	17.2	15.1–19.6
University education or more	1.2	0.7–020
**Is currently studying in prison (*N*** **= 1327)**		
No	71.2	68.8–73.5
Yes	28.8	26.4–31.1
**Number of times incarcerated (*N*** **= 1325)**		
1	31.9	29.3–34.4
2 or +	68.1	65.5–70.6
**Cause of imprisonment (*N*** **= 1327)**		
Drug trafficking	65.6	62.9–68.3
Robbery or theft	17.5	15.5–19.8
Homicide	8.5	7.0–10.3
Criminal Conspiracy	3.1	2.2–4.3
Drug use	3.0	2.1–4.3
Larceny	2.6	1.8–3.7
Armed robbery	2.1	1.3–3.1
Handling stolen goods	1.9	1.2–2.8
Grooming	1.5	0.8–2.5
**Lived on the street (*N*** **= 1326)**		
No	85.8	83.7–87.7
Yes	14.2	12.3–16.3
**Remunerated labor in prison (*N*** **= 1326)**		
No	58.6	55.9–61.4
Yes	41.4	38.6–44.1
**Worked before imprisonment (*N*** **= 1321)**		
No	25.1	21.1–29.5
Yes	74.9	70.4–78.8
**Occupation before prison (*N*** **= 988)**		
Worked with service provision and commerce	31.1	26.8–35.7
Domestic Service	31.8	27.4–36.5
Informal occasional labor (peddler, etc.)	13.1	9.7–17.5
Other	23.8	20.0–28.1
**Before incarceration was the family’s primary source of income (*N*** **= 1325)**		
No	63.5	60.7–66.3
Yes	36.5	33.7–39.3
**Is currently the family’s primary source of income (*N*** **= 1324)**		
No	88.7	86.9–90.4
Yes	11.3	9.5–13.0
**Participates in cash transfer programs (*N*** **= 1311)**		
No	59.4	56.5–62.3
Yes	40.6	37.7–43.5
**Has health insurance (*N*** **= 1319)**		
No	91.0	89.4–92.5
Yes	9.0	7.4–10.5
**Has already been placed in an isolation cell (*N*** **= 1327)**		
No	70.0	67.4–72.5
Yes	30.0	27.1–31.8
**How many share cells (*N*** **= 1324)**		
≤ 5 people	31.9	30.2–33.5
6–10 people	14.2	12.5–16.1
11–19 people	35.0	32.9–37.2
20 or +	18.7	17.3–20.2
**Visitation in prison (*N*** **= 1327)**		
No one	32.3	29.7–35.1
Mother	33.2	30.5–36.0
Brothers	23.7	21.2–26.2
Children	23.4	21–25.9
Spouse or partner	12.1	10.4–14.1
Other relative	8.1	6.7–9.7
Father	6.4	5.1–8.0
Friends	4.6	3.6–5.8
Mother-in-law	2.1	1.5–3.1
**Receives intimate visits in prison (*N*** **= 1315)**		
No	90.0	88.2–91.5
Yes	10.0	8.5–11.8

% = Weighted estimate

Regarding reproductive health, the overwhelming majority reported initiating sex when they were 15 or younger or being forced to have sex (69.5%; 95%IC: 67.0–72.0), and 86.5% (95%IC: 84.4–88.3) had become pregnant at one point in their life, with 81.2% (95%IC: 78.6–83.6) getting pregnant during adolescence. About a third reported an abortion (33.7%; 95%IC: 31.0–36.5), with about a third of these women having 2 or more (28.4%; 95%IC: 22.4–35.8). Almost all the women menstruated (90.1%; 95%CI: 88.9–91.7). The average age of menarche was 12.7 years (SD = 1.81; range 8 to 19; 95%CI: 12.6–12.8); most had children, ranging from 1 to 16 years, with an average of 2.9 children (SD = 1.89, range 0 to 16, 95%CI: 2.7–2.9). Of the children born to these women, (21.9%; 95%IC: 19.3–25.3) were under 5 years of age (Table 2). Considering gynecological examination, (9%; 95%CI: 7.0–10) never had one, and more than half of the prisoners (55.3%; 95%CI: 52.8–57.8) had never undergone preventive testing for cervical cancer in prison. Potential symptoms of STIs were identified in nearly half of the inmates (51.8%; 95%CI: 48.9–54.6), most of whom sought medical attention (84.1%; 95%CI: 80.7–87.0) and reported symptom cure (81.4%; 95%CI: 77.6–84.6) (Table 3).

**Table 2:** Reproductive health characteristics of incarcerated women in Brazil, 2018

Characteristics	%*	95%IC
**Presents menstrual cycle (*N*** **= 1327)**	90.1	88.1–91.7
**Does not present dysmenorrhea (*N*** **= 1198)**	71.8	69.0–74.5
**Has regular menstruation (*N*** **= 1198)**	67.4	64.5–70.3
**Bleeding does not last many days (*N*** **= 1198)**	80.3	77.7–82.7
**Age at first sexual relation (*N*** **= 1308)**		
≤ 15	69.5	67.0–72.0
> 16	30.5	28.0–33.0
**Already been pregnant (*N*** **= 1325)**		
No	13.4	11.6–15.5
Yes	86.5	84.4–88.3
**Age group at first gestation (*N*** **= 1116)**		
Adolescence (10–19 years)	81.2	78.6–83.6
Adulthood (20–59 years)	18.7	16.3–21.3
**Has been pregnant during imprisonment (*N*** **= 1324)**		
No	99.3	98.6–99.7
Yes	0.7	0.3–1.3
**Has had an abortion (*N*** **= 1327)**		
No	66.2	63.4–68.9
Yes	33.7	31.0–36.5
**The abortion was induced (*N*** **= 444)**		
No	85.1	81.4–88.1
Yes	14.8	11.8–18.5
**Number of abortions (*N*** **= 449)**		
01	71.4	66.7–75.7
02	20.3	16.6–24.6
03 or more	8.1	5.8–11.2
**Has children (*N*** **= 1316)**		
No	18.0	15.9–20.3
Yes	81.9	79.6–84.1
**Age of the oldest child (*N*** **= 1282)**		
< 1 year	0.15	0,0–1.0
1–4	6.6	0.53–0.83
5–9	16.7	14.6–19.1
10–17	26.2	23.6–28.9
> 18	50.1	47.2–53.1
**Age of the youngest child (*N*** **= 1272)**		
< 1 year	0.9	0.5–1.7
1–4	21.0	18.7–23.6
5–9	17.8	15.6–20.3
10–17	12.3	10.5–14.4
> 18	47.7	44.8–50.6
**Uses any type of contraceptive (*N*** **= 1311)**		
No	71.4	68.7–73.9
Yes	28.5	26.0–31.3
**Oral contraceptive (*N*** **= 361)**	44.7	39.4–50.1
**Injectable contraceptive (*N*** **= 361)**	37.1	32.0–42.5
**IUD (N = 361)**	4.0	2.0–8.1
**Condom (361)**	30.0	25.5–34.9
**Has/had free access to condoms (*N*** **= 1318)**		
School	8.4	6.8–10.3
Prison	15.0	13.2–17.0
Work	2.3	1.6–3.4
Public service	54.4	51.7–57.2
Private service	3.4	2.4–4.6

% = Weighted estimate

**Table 3:** Access to healthcare service of women incarcerated in Brazil, 2018

Characteristics	%	95% IC
**Last time that had a gynecological examination (*N*** **= 1277)**		
Never had one	9.0	7.0–10
In the past 03 years	76.8	74.0–79.0
4 to 5 years	7.2	5.9–8.7
More than 5 years	6.7	5.4–8.4
**Has undergone preventive cervical cancer examination in prison (*N*** **= 1190)**		
No	55.3	52.8–57.8
Yes	44.6	42.1–47.1
**Had a mammogram during imprisonment (*N*** **= 1190)**		
No	85.6	83.3–87.7
Yes	14.3	12.2–16.6
**When last had a mammogram (*N*** **= 1319)**		
Never had one	72.9	70.3–75-5
In the past 3 years	19.9	17.7–22.4
In the past 4–5 years	3.9	2.9–5.2
More than 5 years	3.1	2.0–4.3
**These exams (prevention and mammogram) were offered in prison (*N*** **= 1190)**		
Yes	47.4	44.9–5.0
No	52.2	50. 0–55.0
**At the last gynecological examination, did a Pap smear (*N*** **= 1111)**		
No	16.0	13.9–18.3
Yes	83.9	81.6–86.0
**Had signs and symptoms suggestive of STI in life (*N*** **= 1305)**		
No	48.2	45.4–51.1
Yes	51.8	48.9–54.6
**Types of symptoms in the genital region (*N*** **= 636**)		
Vaginal discharge	92.8	88.6–95.6
Sores	14.1	10.6–18.4
Small blisters	10.0	6.9–14.2
Warts	11.2	7.0–16.8
**When at symptom presentation, underwent treatment (*N*** **= 631)**		
No	18.5	15.5–21.8
Yes	81.4	78.1–84.8
**Which people did you seek to resolve the problem (*N*** **= 506)**		
Doctor	84.1	80.7–87.0
Pharmacist	2.6	1.0–4.0
Nurse	9.8	7.0–12.6
Public health agent	3.7	2.2–5.9
Prison guard	4.5	3.0–6.7
I did not seek anyone, I treated myself	2.0	1.0–3.8
**Took any medication for the symptoms suggestive of STI (*N*** **= 505)**		
I took the medication, but was not cured	10.1	7.7–13.1
Yes, I was cured	81.4	77.6–84.6
I don’t know if I was cured, but the symptoms disappeared	8.5	6.2–11.3
**After feeling these problems, received which orientations (*N*** **= 376)**		
Use condoms regularly	68.4	63.3–73.2
Inform partner that you were ill	39.3	34.2–44.7
Test for HIV	31.7	26.8–37.0
Test for Syphilis	24.3	19.8–29.3

% = Weighted estimate

## Discussion

The findings of the present study show that the majority of the incarcerated women in the country are young mothers of reproductive age. These results imply the urgent need for prisons to adapt to the specific demands of these women and to offer them a full range of reproductive health services, be they preventive or curative. Many of these women are in the economically active period of their lives. Most worked before being incarcerated and just over 1/3 were the main source of the family income. This income is lost to the families, adding to the cycle of poverty. These women started in deficit to begin with, given the substantial gender-related disparities in income in Brazil [14, 15]. The scenario of the feminization of poverty is evidenced in this study where many women were responsible for Family income before incarceration.

Approximately half of the women studied reported having given birth to, children or adolescents under age 19, and 40% of these were under 10 years old at the time of the study. In 2016, a law was passed in Brazil aimed at guaranteeing pregnant women and mothers of children under the age of 12 the opportunity to serve their sentence at home, attempting to ensure greater support for the child. To realize this benefit, the woman must not have committed a crime against her children, not be part of a criminal gang, and not be a repeat offender [16]. The law would guarantee maternal presence in the family and would contribute to women being able to work, since around 37.3% of Brazilian women in general, and 87.4% of single mothers with children are heads of household [17]. Nevertheless, due to recidivism or being convicted of drug traffiking, many women do not meet these criteria, and for those that do, having no evidence of fixed permanent address, or being unable to present documentation, such as birth records or demonstration of children living with them or being accused of risky conduct that threatens the child from a spouse, ex-spouse, or other family member excludes them from the benefit [18, 19]. For these women, being pregnant or having young children means that their children will be in the care of family members, neighbors, or in state custody [20], increasing the rate of institutionalized children [21]. For the children of imprisoned mothers, the absence of their mother can be profound in both developmental terms and in relation to peers, neighbors and the community [21, 22]. The narrative of maternal status, relations to ones’mother and personhood in Brazil has broad repercussions. The distancing created by incarceration affects the health status of the mother and can have disastrous consequences for children, during childhood, adolescence and adulthood [22, 23]. Knowing the effects on their children’s lives generates mixed feelings in women. After all, many want their children nearby and wish to be there during critical moments. On the other hand, they do not want them exposed to the environment of Brazilian prisons, which are certainly unsuitable for children, even without the strict body cavity search that takes place prior to family visits [6].

This study showed something of the reality of Brazilian women in prison. The large number of women of color and women of low income in prison marks social and class differences, and makes the judicial system suspect. The large number incarcerated for drug crimes speaks more to society’s addictions and the predatory behavior of partners than individual moral qualities. Incarceration is associated with family disintegration, poor social conditions, low education, little expectation of social reintegration, and the difficulty of improving their lives [24]. Given these circumstances, women find themselves without expectations of change in their social and financial conditions after prison, favoring recidivism. Approximately 68.1% of the women are repeat offenders, with drug trafficking as the main reason for prison. The motivations for drug trafficking can be difficult to parse. Women’s involvement in trafficking includes not realizing that it is a “crime”, but rather slightly illegal work needed to support the family encouraged by a partner. Sometimes women are motivated by disappointment in relationships, or are users and need to support their habit [25, 26]. Motivations can reach the level of novelistic pathos, with prison reported in one trial account an attractive option for a HIV+ women from a country that does not provide ARVs, since treatment is purportedly guaranteed in Brazilian prisons [27, 28].

The abandonment of family members and partner/spouse while in prison may also be a factor in health in prisons. It induces loneliness and may contribute to the emergence of psychological disorders and dependence on alcohol and illicit drugs, as well as hindering social reinsertion [11]. Around 32% of women prisoners do not receive visits in prison. This percentage is much higher for intimate visits, 90% do not receive an intimate visit, although intimate visits are legally guaranteed for men and women [29]. However, such rights are hindered and/or neglected for women. Approximately 52% of female prisoners have partners who are also imprisoned, a fact that renders intimate visits even more difficult [30]. In order for the female inmate to go to another prison unit, for the intimate visit to her partner, some conditions such as police escort and prior scheduling are required [28], making us question whether, in view of such bureaucracies and the current prison situation in Brazil, this right is really guaranteed. Travel when possible, is generally carried out by women, with men not traveling to meet with their partners. Intimate visits should be held in a suite where the couple can stay for up to 2 h, bimonthly. However, most prisons do not have this space. Finally the benefit is granted only to those who can prove consensual union or who are married [28].

Female prisoners are abandoned in every way. Partner abandonment can also contribute to poor adherence to contraceptive methods (28.5%), with oral contraceptives (44.7%), injectable contraceptives (37.1%) and condoms (30%) being the most frequently utilized methods. In the prison system, only 15% of the women reported receiving condoms for free. As for condom use, 69.9% of the women denied using them. This figure may be less alarming taking into consideration the difficulty of negotiating with their partners, restricted access, and high reported levels of same sex relationships [31, 32].

When women use a contraceptive method, they are seeking to prevent another unwanted pregnancy or reduce the risk of abortion, with oral and injectable contraception being the most widely used and accessible resource within and outside the prison system [33, 34]. In Brazil, the frequency of abortion is elevated, although condemnation of abortion is almost universal. Addressing abortion is made difficult because voluntary abortion is illegal, and the topic is treated from a religious and moral perspective. For the majority of women, however, voluntary abortions are performed in a two-stage fashion. First, women initiate abortion outside of hospital, and then, once initiated, or following abortion, attend the hospital for completion. This procedure leads to a high rate of complications [35]. In prisons, women who have abortions tend to be isolated and expelled from their cells and are sent to a specific “safe” place, which, in male prisons, is destined for rapists and/or “child killers” [20].

Prisoners are poor women coming from marginal and precarious living environments who may not have had access to quality health care. This renders it a suitable locus for the implementation of preventive strategies, as well as the provision of basic health services, including early detection of breast cancer and cervical cancer prevention [24]. For many women, incarceration is the only opportunity to gain access to health services [33]. For the year 2018, it is estimated that 59,700 new cases of breast cancer and 16,360 new cases of cervical cancer occur in the female population of Brazil. Gynecological examination and cervical cancer screening are an effective strategy for the early detection of many diseases [35]. The last cervical cancer screening for 76.8% of the women in our sample took place 3years previously. While the recommendation for cervical cancer screening is every 3 years if the last two annual exams were normal [36] there is reason to believe that women in prison represent a higher risk population. In several studies, women aged 40 and older in prison demonstrated a risk of developing cervical cancer 4 to 5 times higher compared to the general population [37, 38]. Such vulnerability intensifies when early sexual debut occurred, the individuals are smokers, and they have prolonged use of oral contraceptives [39].

Regarding mammograms in prison, 42,7% of women over the age 50 have never done reported the exam. These results are similar to the study conducted by Audi (2016), [24], who identified low prevalence of both cervical cancer screening and mammograms. Several factors are associated with low number of mammograms, including being single, having little education, being of low social class, lack of knowledge of prevention methods, and lack of access to free services [40]. Due to the high percentages of young women in prison, there are great consequences in not carrying out the appropriate breast cancer screening program [24]; screening has been demonstrated to be an essential strategy for reducing breast cancer mortality in women aged 40 to 49 years old [41].

In addition to screening for breast and cervical cancers, STI symptoms need to be investigated during consultations. The occurrence of other STIs, associated with the profile of women and men in prison and low levels of condom use, potentiate the risk for HIV/AIDS, contributing to a serious public health problem within and outside the prison system, including high risk behavior post-release [42–44]. Asymptomatic STIs, such as chlamydial infections (75%), gonorrhea (50%), and many cases of HIV and syphilis occur are transmitted and may lead to complications such as sterility [45]. Prison health systems can do much to reduce health inequity by treating this population [46–48].

## Conclusion

The profile of female prisoners in Brazil shows that the reproductive health of women in the prison system is a public health challenge. They represent a long history of gender violence and abuse that starts early in their lives. Moreover, the incarceration of women has repercussions on family, especially when children and teenagers are involved. Maternal absence at these stages of life can create exactly the kinds of behavior that incarceration was meant to reduce. These women are also routinely denied basic health rights such as house-arrest. Rather than a simple benefit, house-arrest, especially in combination with appropriate social services could go far to reduce the consequences of incarceration and also reduce individual and state debt.

The health of incarcerated women constitutes both a challenge and an opportunity for public health. Overall, the poor women of color who constitute the female prison population in our sample and in Brazil are victims; perpetrators in only a small sense. They get very little from their prison experience and are thrust back into the circumstances that landed them in prison in the first place. While prisons have little or nothing in the way of resources and opportunities to change the environments these women are released into, at least they could return healthier women, more knowledgeable and better educated, to their families and these circumstances. That would at least be a demonstration of understanding and consideration.

## Supplementary Information

**Additional file 1.** Complete data collection instrument.

## Data Availability

In accordance with the submission rules of this journal, I declare that, if necessary, the sending of the database, it may be requested through the e-mail of the professor and coordinator of the project PHD. Ligia Kerr, through her e-mail. Contact: PHD Ligia Kerr E-mail: ligiakerr@gmail.com

## Abbreviations

ACASI: Audio Computer-Assisted Self-Interviewing
ARV: Antiretrovirals
CEP: Research Ethics Committee
CNS: National Health Council
FD: Federal District
HIV / AIDS: Human Immunodeficiency Virus / Human Immunodeficiency Syndrome
SPSS: Statistical Package for the Social Sciences
STATA: Statistical Software
STI: Sexually Transmitted Infection
