# Correction to: Behind bars: the burden of being a woman in Brazilian prisons

**DOI:** 10.1186/s12914-020-00250-y

**Published:** 2020-12-14

**Authors:** Priscila França de Araújo, Ligia Regina Franco Sansigolo Kerr, Carl Kendall, George W. Rutherford, David W. Seal, Roberto da Justa Pires Neto, Patrícia Neyva da Costa Pinheiro, Marli Teresinha Gimeniz Galvão, Larissa Fortunato Araújo, Francisco Marto Leal Pinheiro, Ana Zaira da Silva

**Affiliations:** 1 Department of Public Health, Federal University of Ceara, Professor Costa Mendes, 1608 - Didactic Block, 5th floor Neighboor Rodolfo Teófilo, Fortaleza, Ceará 60.430-140 Brazil; 2 School of Public Health and Tropical Medicine Tulane University, New Orleans, USA; 3 University of California, San Francisco, USA

**Correction to: BMC Int Health Hum Rights (2020) 20:28**

**https://doi.org/10.1186/s12914-020-00247-7**

It was highlighted that in the original article [1] one affiliation was omitted from author Carl Kendall. This Correction article shows the correct affiliations. The original article has been updated.
