# Supplementary material for: Behind bars: the burden of being a woman in Brazilian prisons
Source: BMC Int Health Hum Rights. 2020 Oct 29;20:28. doi: 10.1186/s12914-020-00247-7 (PMC7594946; doi:10.1186/s12914-020-00247-7)
Supplement: Supplementary file 1 — Additional file 1. Complete data collection instrument. [file 12914_2020_247_MOESM1_ESM.docx]

study.id Subject ID Search

study.id.2 Suj ID confirmation

PR.A.2. Date of Birth (dd / mm / yyyy)

{"validation": "dateMMYYY"}

PR.A.3. Date of Entry into the Prison System (mm / yyyy)

{"validation": "custom [dateMMYYYY], funcCall [mesAnoMenorQue [pr_a_3]", "mask": "99/9999"}

PR.A.4. Date of admission to this prison unit (mm / yyyy)

{"type": "introduction", "name": "pr_componente_a_1"}

Subcomponent: Inclusion criteria

PR.A.5. What is your color or race?

[] Black

[] Brown

[] White

[] Yellow

[] Indigenous

[] Other

[] NSNQR

PR.A.1.1. What is your level of education?

[] Illiterate

[] 1st to 3rd grade of elementary school

[] 4th to 7th grade of elementary school

[] Complete elementary school (finished 8th or 9th grade)

[] 1st or 2nd year of high school

[] Complete high school (finished 3rd high school or 3rd scientific year)

[] Incomplete higher

[] Graduated

[] Other

[] NSNQR

PR.B.1. What is your current marital status?

[] Single and without partner or steady partner

[] Has a steady partner

[] Has a fixed partner

[] Married or stable union

[] Separate

[] Divorced or divorced

[] Widow

[] Other

[] NSNQR

PR.B.2. What is your religion or religious belief?

[] I have no religion or belief

[] Catholic

[] Evangelical

[] Spiritist

[] Other

[] NSNQR

PR.B.3. What was your occupation before you were arrested?

[] Didn't work

[] Senior government official, officer, manager or senior company employee

[] Higher education professional

[] Professional arts

[] Mid-level professional or technician

[] Administrative services worker

[] Service and commerce worker

[] Domestic service worker

[] Agricultural worker, hunting and fishing forestry

[] Manual worker (production of industrial goods and services)

[] Manual construction worker

[] Manual repair and maintenance worker

[] Member of the armed forces, police and military fireman

[] Poorly specified occupations of informal work (traveling, valet, car guard, etc.)

[] Other

[] NSNQR

PR.B.4. Have you ever been a homeless person?

[] Yes

[] No

[] NSNQR

PR.B.5. Are you currently studying here in prison?

[] No

[] Yes

PR.B.13. How many times have you been arrested?

[] 1

[] 2

[] 3

[] 4

[] 5

[] 6

[] More than 6

[] NSNQR

PR.E.2. Do you still menstruate?

[] No

[] Yes

[] NSNQR

PR.E.3. How is your period? (CHECK HOW MANY OPTIONS ARE TRUE)

[] I no longer menstruate @exclusive

[] Normal, comes every month and doesn't usually bother >> PR.E.5

[] Irregular, there is next month >> PR.E.5

[] Bleeding lasts for many days >> PR.E.5

[] I have a lot of colic >> PR.E.5

[] Other >> PR.E.5

[] NSNQR >> PR.E.5

PR.E.4. If you don't menstruate anymore, how old were you when you stopped menstruating?

PR.E.6. How old were you when you first got pregnant?

PR.E.7. How many times have you become pregnant?

PR.E.8. Have you ever had an abortion or abortion?

[] No >> PR.E.12

[] Yes

[] NSNQR >> PR.E.12

PR.E.9. How many abortions or abortions have you had?

PR.E.17. What contraceptive method do you use? (Check as many options as are true)

[] Oral contraceptives

[] Injectable contraceptives

[] IUD

[] Diaphragm

[] Condom

[] Other

[] NSNQR

PR.E.18. When was the last time you had a gynecological exam?

[] Never did >> PR.E.20

[] In the past 3 years

[] 4-5 years ago

[] Over 5 years ago

[] NSNQR >> PR.E.20

PR.E.23. Have you had any of these symptoms, in your life? (CHECK HOW MANY OPTIONS ARE TRUE)

[] Runny

[] Wound on the private parts

[] Small bubbles in the private parts

[] Warts on the private parts

[] I never had any of these symptoms >> pr_componente_f @exclusivo

[] NSNQR >> pr_componente_f

R.E.18. When was the last time you had a gynecological exam?

[] Never did >> PR.E.20

[] In the past 3 years

[] 4-5 years ago

[] Over 5 years ago

[] NSNQR >> PR.E.20

PR.E.19. Thinking about the last time you took the gynecological exam, did you take the exam called Pap smear, where the doctor or nurse collected material for preventive cancer exam?

[] No

[] Yes

[] NSNQR

PR.E.20. When was the last time you had a mammogram?

[] Never did

[] In the past 3 years

[] 4-5 years ago

[] Over 5 years ago

[] NSNQR

{"type": "checkbox"}

PR.E.21. In the prison where you are or another one that has passed, were any of these exams offered? (CHECK HOW MANY OPTIONS ARE TRUE)

[] Cervical cancer prevention exam

[] Mammography exam

[] These exams were never offered in prison

[] NSNQR
